# The Upregulation of Regenerative Activity for Extracellular Vesicles with Melatonin Modulation in Chemically Defined Media

**DOI:** 10.3390/ijms232315089

**Published:** 2022-12-01

**Authors:** Jun Yong Kim, Won-Kyu Rhim, Jiwon Woo, Seung-Gyu Cha, Chun Gwon Park, Dong Keun Han

**Affiliations:** 1 Department of Biomedical Science, CHA University, Seongnam 13488, Republic of Korea; 2 Department of Biomedical Engineering, SKKU Institute for Convergence, Sungkyunkwan University (SKKU), Suwon 16419, Republic of Korea; 3 Intelligent Precision of Healthcare Convergence, SKKU Institute for Convergence, Sungkyunkwan University (SKKU), Suwon 16419, Republic of Korea

**Keywords:** extracellular vesicle (EV), chemically defined media (CDM), melatonin, microRNA (miRNA), tissue regeneration

## Abstract

Extracellular vesicles (EVs) derived from human mesenchymal stem cells (hMSCs) have been widely known to have therapeutic effects by representing characteristics of the origin cells as an alternative for cell-based therapeutics. Major limitations of EVs for clinical applications include low production yields, unknown effects from serum impurities, and relatively low bioactivities against dose. In this study, we proposed a cell modulation method with melatonin for human umbilical cord MSCs (hUCMSCs) cultured in serum-free chemically defined media (CDM) to eliminate the effects of serum-derived impurities and promote regeneration-related activities. miRNAs highly associated with regeneration were selected and the expression levels of them were comparatively analyzed among various types of EVs depending on culture conditions. The EVs derived from melatonin-stimulated hUCMSCs in CDM (CDM mEVs) showed the highest expression levels of regeneration-related miRNAs, and 7 times more hsa-let-7b-5p, 5.6 times more hsa-miR-23a-3p, and 5.7 times more hsa-miR-100-5p than others, respectively. In addition, the upregulation of various functionalities, such as wound healing, angiogenesis, anti-inflammation, ROS scavenging, and anti-apoptosis, were proven using in vitro assays by simulating the characteristics of EVs with bioinformatics analysis. The present results suggest that the highly regenerative properties of hUCMSC-derived EVs were accomplished with melatonin stimulation in CDM and provided the potential for clinical uses of EVs.

## 1. Introduction

Mesenchymal stem cell (MSC)-derived extracellular vesicles (MSC-EVs) have been utilized to treat a wide range of diseases such as coronavirus disease 2019 (COVID-19) [1], graft-versus-host-disease (GVHD) [2], and osteoarthritis (OA) [3], while reflecting the parental cells’ properties. In a recent study on COVID-19 treatment, EVs showed the therapeutic effects to improve patient survival with regulating lung infiltrations of neutrophil and macrophage, as well as pulmonary edema [4]. The MSC-EVs administered intravenously in acute GVHD murine models increased animal survival and controlled cytokine production, with an increase in IL-10 production and an downregulation of TNF-α, IL-17, and IL-2 expression [5]. Additionally, the intra-articular injection of EVs was demonstrated to have both preventive and therapeutic advantages for OA murine models in lots of studies [6]. With an increasing number of trials investigating their uses in the treatment of various diseases, the cell culture condition for EVs has been considered as a critical factor to control production yield, purity, and functionalities for clinical use [7].

In our previous study, culturing cell with chemically defined media (CDM) has been proven to boost EV production with excluding FBS-derived contaminants for high purities [8]. Unlike the starvation conditions, as a gold standard to exclude FBS during EV isolations, cell culture with CDM maintained healthy cells during EV isolations and enabled cells to release anti-inflammatory cytokines [9]. Moreover, various stimulation strategies have been investigated to enhance EV functionalities, such as 3D culture [10], hypoxia [11], and cell priming [12]. Cell priming is a stimulation method with pro-inflammatory factors, with the most common being TNF-α and IFN-γ (TI), to improve EV functionality by encouraging cells to release substances to recover inflammation [13]. However, TI priming may not only cause an increase in anti-inflammatory factors but pro-inflammatory cytokine within the EV. To enhance the regeneration-related activities of EVs without pro-inflammatory factors in EVs, melatonin has been applied for cell modulations due to versatile properties of melatonin, including enhancements of cell proliferation [14], angiogenesis [15], and anti-fibrosis effects [16]. Yea et al. have reported that EVs from melatonin-stimulated adipose mesenchymal stem cells (ADMSCs) improved kidney function by regulating inflammation and fibrosis in a chronic kidney disease (CKD) murine model [17].

In this study, to maximize the therapeutic activities of human umbilical cord MSC (hUCMSC)-derived EVs, we comparatively analyzed the characteristics of four different types of hUCMSC-derived EVs: EVs from hUCMSCs in starvation (STV EVs), EVs from melatonin-stimulated hUCMSCs in starvation (STV mEVs), EVs from hUCMSCs in CDM (CDM EVs), and EVs from melatonin pretreated hUCMSCs in CDM (CDM mEVs). To predict and distinguish bioactivities for various types of EVs, bioinformatics analysis was performed using miRNA in EVs. The expression levels of representative miRNAs associated with tissue regeneration were compared and the targeting genes of miRNAs were identified using miRWalk and Target Scan 8.0. In addition to the relevance of miRNA targeting genes, the functionalities of EVs were predicted based on the results of gene ontology (GO) and the Kyoto Encyclopedia of Gene and Genomes (KEGG) analysis from the DAVID resources. With expectations from the results of bioinformatics analysis, maximized regeneration-related functionalities of CDM mEVs were demonstrated through various cell-based assays. The comparative analyses have suggested the optimum strategy to modulate cells for significant improvements in regeneration-related activities for future applications of MSC-derived EVs for kidney regenerations.

## 2. Results and Discussion

### 2.1. Characterization of hUCMSC-Derived EVs Depending on Culture Conditions

**Figure 1:**
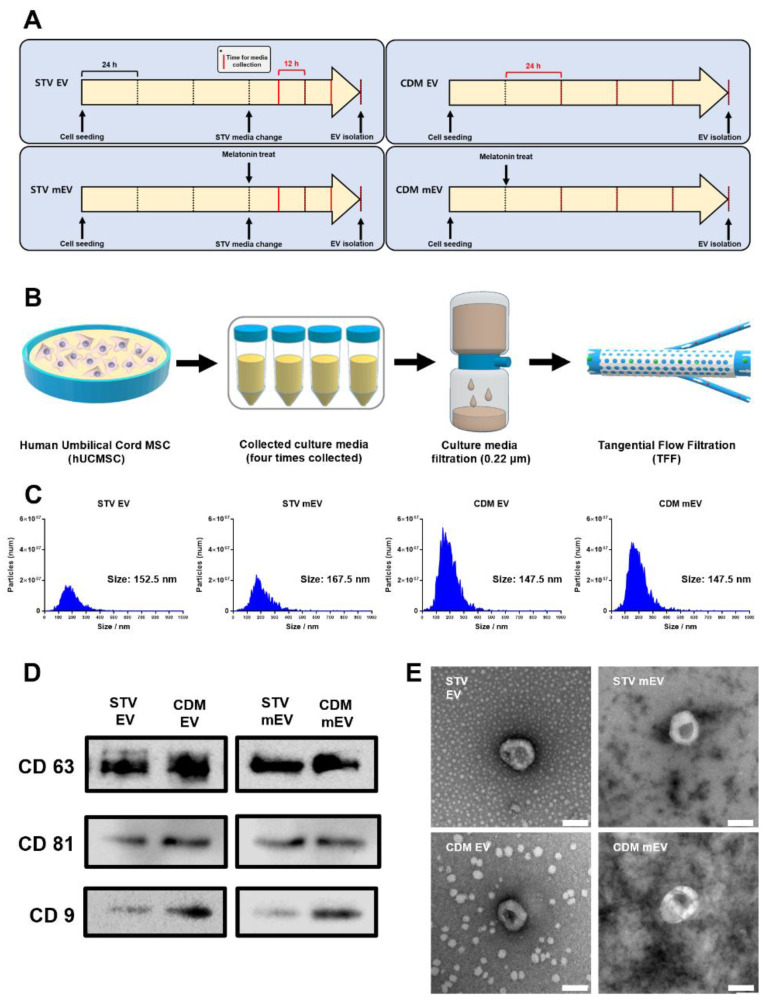
The processes for EV isolations and characterizations of EVs derived from hUCMSCs in various culture conditions. (**A**) Time plans for cell culture and EV isolations. (* The time for media collection was presented with the vertical red line.) (**B**) The illustration for processes of EV isolation. (**C**) ZetaView analysis for the number of total particles and size of STV EVs, STV mEVs, CDM EVs, and CDM mEVs. (**D**) Western blot analysis for representative surface markers of EVs (CD63, CD81, and CD9). (**E**) The morphology of EVs characterized by TEM. Scale bars equal to 100 nm.

The chemically defined media (CDM; CellCor^TM^ CD MSCs) have been shown to improve EV production and functionality in our previous work [9]. To maximize the regeneration-related bioactivities of hUCMSC-derived EVs, we additionally stimulated cells with a biochemical component. Cell priming is a popular method used for stimulating cells with various pro-inflammatory components, such as curcumin, LL37, IFN-γ, TNF-α, and their combinations, to induce the secretion of useful factors during recovering inflammatory conditions [13]. Unlike the priming methods with pro-inflammatory factors, cell stimulation with melatonin has expected to impact the regeneration of tissues by maintaining cells healthy because melatonin is well known for immune regulatory functions, such as reducing apoptotic and necrotic changes, inflammatory cell infiltration, and tissue fibrosis after injury by suppressing fibrotic effects in wounded cells [18,19]. Furthermore, several types of researches demonstrated that melatonin had effects on disorders including hindlimb ischemia [20], hepatic ischemia [21], and kidney ischemia [22] by utilizing the profitable properties of melatonin. To prove the synergistic activities of EVs preconditioned with CDM and melatonin, four different cell culture conditions were set, as shown in Figure 1A. Unlike cell culture with CDM, the cell culture media were replaced with serum-depleted media during EV isolations to exclude serum impurities in starvation condition of normal media. The melatonin was pretreated to cells during culturing cells and conditioned media were collected four times to maximize the production yield of EVs. Compared to the isolation of EVs from starvation conditioned in normal media, they have the advantages of being able to obtain EVs, in which serum components are excluded for a long period of culture, and that the duration of melatonin treatment could be adjusted, in anticipation of higher productivity and functionalities. The tangential flow filtration (TFF) method was performed to isolate various types of EVs (STV EVs, STV mEVs, CDM EVs, and CDM mEVs) from collected conditioned media (Figure 1B). The number and size of EVs were evaluated using MONO ZetaView^®^ (Figure 1C). Pretreatments of melatonin did not have a significant effect on the production yield of EVs; however, a production yield of EVs that was two times higher was achieved with CDM compared to STV, in correlation with our previous results [9]. The representative EV markers (CD63, CD81, and CD9) were validated using Western blot analysis in accordance with MISEV 2018 guidelines for further verification (Figure 1D) [23], and a cup-shaped typical EV morphology [24] was confirmed by TEM analysis (Figure 1E).

### 2.2. The Expectations for the Functionality of EVs through Comparative Analysis on Internal miRNA of EVs

**Figure 2:**
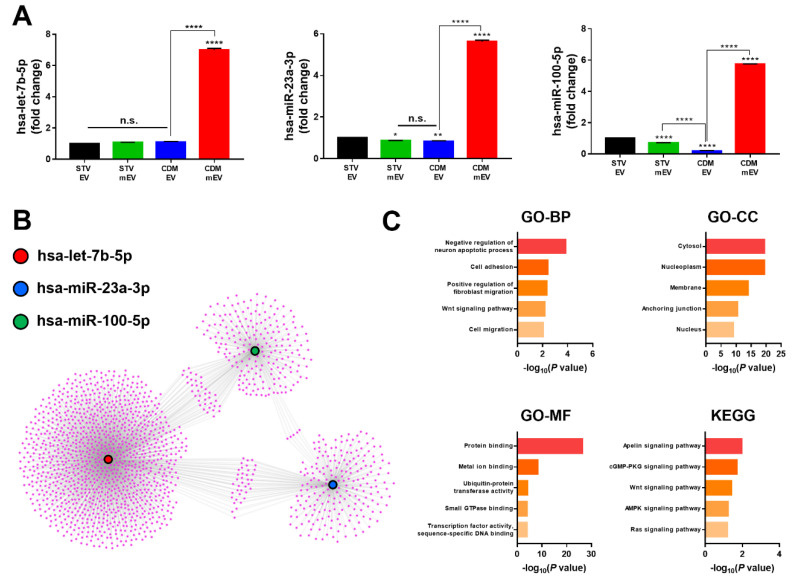
The analysis of regeneration-related miRNAs in EVs. (**A**) The internal miRNA expression levels of let-7b-5p, miR-23a-3p, and miR-100-5p in EVs determined by RT-qPCR. (**B**) The gene cluster and networks of internal miRNAs in EVs. (**C**) The DAVID analysis of gene ontology (GO) and Kyoto Encyclopedia of Genes and Genomes (KEGG) with target genes of miRNAs associated with the regeneration effect. Values are presented as mean ± SD (*n* = 3) and statistical significance was obtained with one-way analysis of ANOVA with Tukey’s multiple-comparison post-test (* *p* < 0.05; ** *p* < 0.01; **** *p* < 0.0001).

The paracrine effects of EVs on cells regulate a broad range of functions, and they occur through the transportation of genetic materials, including mRNAs and miRNAs [25]. Three types of miRNAs, such as let-7b-5p, miR-23a-3p, and miR-100-5p, have been demonstrated to control regeneration-related processes, including angiogenesis, cell proliferation, migration, and others [26,27,28], and exert superior regeneration effects on the kidney [17], bone [29], and cartilage [30], respectively. We selected these miRNAs as some of the representative miRNAs used for regenerations, and quantitatively compared the expression levels of them in STV EVs, STV mEVs, CDM EVs, and CDM mEVs. (Figure 2A). The CDM mEVs contained a greater abundance of all types of miRNAs than the other groups. Based on the results of RT-qPCR for three representative miRNAs, we hypothesized that the effects of melatonin stimulations to culturing cells were upregulated only in the CDM condition, resulting in the greatest effects on tissue regenerations. To anticipate the upregulated functionalities of CDM mEVs, bioinformatics analysis based on the expression of internal miRNAs was performed. The miRWalk was used to predict the target genes for the three miRNAs (let-7b-5p, miR-23a-3p, and miR-100-5p), and miRNet 2.0 was used to establish their network (Figure 2B). Using the database for annotation, visualization, and integrated discovery (DAVID), the cellular responses for CDM mEVs were simulated with the target genes of the miRNAs upregulated in CDM mEVs. Biological processes (BPs), cellular components (CCs), molecular functions (MFs), and signaling pathways regulated by the facilitating miRNAs were validated using gene ontology (GO) and the Kyoto Encyclopedia of Genes and Genomes (KEGG) pathway (Figure 2C). The GO-biological process (GO-BP) displayed a “Negative regulation of neuron apoptotic process”, “Cell adhesion”, a “Positive regulation of fibroblast migration”, and “Cell migration”. Through the result of GO-BP, it was estimated that the CDM mEVs had improved anti-apoptosis, cell migration, and cell adhesion properties. Furthermore, it was speculated that CDM mEVs had anti-inflammatory and angiogenesis effects by regulating the “Wnt signaling pathway”, as shown in KEGG analysis [31,32]. The GO-CC and GO-MF terms indicated that three types of miRNAs (hsa-let-7b-5p, hsa-miR-23a-3p, and hsa-miR-100-5p) were related with “Cytosol” and “Nucleoplasm” in cellular components, and “Protein binding” and “Metal ion binding” in molecular functions. The KEGG pathway exhibited the “Apelin signaling pathway”, the “cGMP-PKG signaling pathway”, the “Wnt signaling pathway”, the “AMPK signaling pathway”, and the “Ras signaling pathway”. All these signaling pathways are associated with angiogenesis and anti-inflammation, which are critical factors to control regenerative activities [32,33,34,35,36]. In other words, the Apelin signaling pathway is linked with anti-inflammation, anti-apoptosis, and ROS scavenging [37,38]. Furthermore, the Wnt signaling pathway has been promoted during tissue regeneration by increasing stem cell activity in the diverse organs [39]. Moreover, the AMPK signaling pathway is related to anti-inflammation and antioxidant effects [40], and the Ras signaling pathway was also associated with cell proliferation, differentiation, and wound healing [41,42]. Considering the GO and KEGG pathway analyses, we hypothesized that CDM mEVs would indicate the greatest regeneration effect compared to other EVs.

### 2.3. Angiogenesis and Wound-Healing Effects of EVs

**Figure 3:**
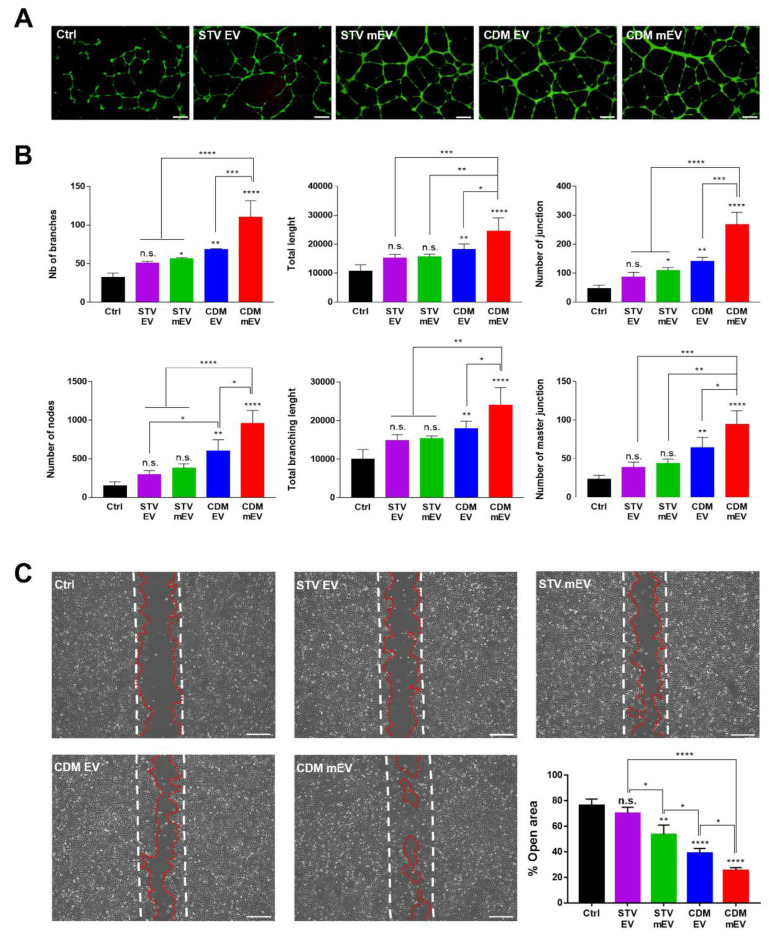
The angiogenesis and wound-healing activities of EVs. (**A**) The representative images of angiogenic effect of EVs by tube formation assay in HCAECs. Scale bars equal to 400 μm. (**B**) Analysis of angiogenesis-related parameters. (**C**) The representative images of cell migration assays to demonstrate wound-healing activities of EVs in HK2 cells. Scale bars equal to 400 μm. Analysis with Image J, values are presented as mean ± SD (*n* = 3) and statistical significance was obtained with one-way analysis of ANOVA with Tukey’s multiple-comparison post-test (* *p* < 0.05; ** *p* < 0.01; *** *p* < 0.001; **** *p* < 0.0001).

Following on the bioinformatics analysis of internal miRNAs in EVs, the CDM mEVs were expected to show highly active angiogenic properties. The angiogenesis was correlated to all pathways in the KEGG that were analyzed via our bioinformatics approach. We executed a tube formation assay to validate the angiogenic effects of EVs. The results of the tube formation assay show that the complexity of tube morphology was mostly promoted in the group of CDM mEVs (Figure 3A). In addition, the CDM mEV exhibited the highest level in the various parameters of angiogenesis assessment, such as the number of branches, the total length, the number of junctions, the number of nodes, the total branching length, and the number of master junctions. It was estimated that the angiogenic properties of MSC-derived EVs were upregulated with containing various angiogenic factors, VEGF, angiopoietin-1, FGF, and EGF in the cell culture condition of CDM [9], facilitated with an additional melatonin-based stimulation [43]. The wound healing could be accelerated by a robust and vigorous angiogenic response that supplies oxygen and nutrients for cellular proliferation, migration, and metabolic processes [44]. We also hypothesized that CDM mEVs might have a significant wound-healing effect based on the bioinformatics analysis of internal miRNAs in EVs, especially in GO-BP terms relating to the “Positive regulation of fibroblast migration” and “Cell migration”. The Wnt and Ras signaling pathways in the KEGG have known to also be related to tissue regeneration and wound healing, respectively. In these regards, a cell migration assay was conducted to demonstrate the wound-healing efficacy of EVs (Figure 3C). The results reveal that CDM mEVs showed the highest wound-healing effect and CDM mEVs could contribute to intricately interacting angiogenesis and wound healing due to the orchestrated effects of CDM and melatonin by regulating several signaling pathways of cells.

### 2.4. Anti-Inflammation Effects of EVs

**Figure 4:**
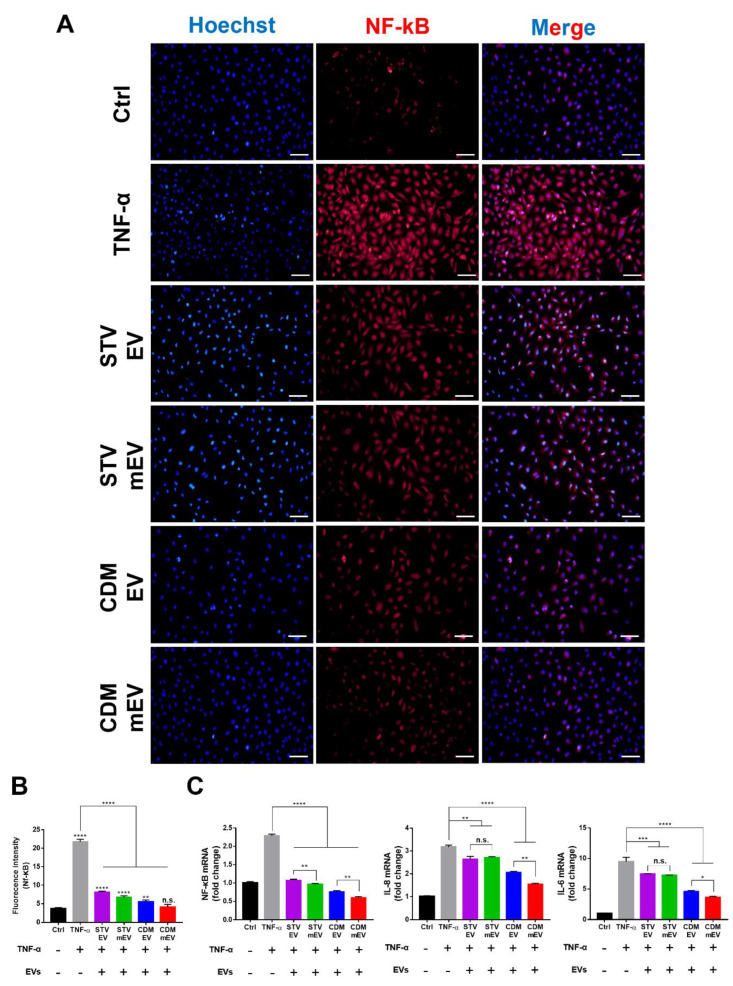
The anti-inflammation effects of EVs in HK2 cells. (**A**) The immunocytochemistry (ICC) images and (**B**) quantification data of the anti-inflammation effect of EVs using Image J. Blue and red indicate nuclei and NF-κB, respectively. Scale bars equal to 100 μm. (**C**) The quantitative analysis for gene expression levels of inflammation-related factors (NF-κB, IL-8, and IL-6) using RT-qPCR. Values are presented as mean ± SD (*n* = 3) and statistical significance was obtained with one-way analysis of ANOVA with Tukey’s multiple-comparison post-test (* *p* < 0.05; ** *p* < 0.01; *** *p* < 0.001; **** *p* < 0.0001).

Inflammation has been implicated in a variety of disorders, including COVID-19-induced sepsis [45], chronic kidney disease (CKD) [17], acute kidney injury (AKI) [46], and others. The anti-inflammatory activities of MSC-derived EVs have been proven [47,48], and we also expected to observe the anti-inflammation properties of EVs from the results on the relations between Apelin and AMPK signaling pathways in the KEGG bioinformatics analysis. To compare the anti-inflammation effects of EVs derived from hUCMSCs in various culture conditions, the scavenged level of NF-κB was monitored using immunocytochemistry (ICC) and the quantification results were evaluated from the fluorescence intensities with Image J (Figure 4A,B). The TNF-α-treated HK-2 showed the high intensity of NF-kB, i.e., the acute inflammations, whereas the intensity of NF-κB was reduced in all EV-treated groups, particularly with melatonin stimulations in CDM. In correlation with the results of ICC, the gene expression levels of pro-inflammatory cytokines, NF-κB, IL-8, and IL-6, dramatically reduced in CDM mEVs (Figure 4C). Interestingly, while melatonin did not alter the anti-inflammatory effects of EVs in starvation conditions, it upregulated the anti-inflammatory activity of EVs in CDM. These results are consistent with our previous results in which the expression of intracellular inflammation-related factors increased by insufficient nutrient supply during the starvation process, suggesting that the effect of melatonin is negligible in this condition [9]. The differences in the internal miRNA of EVs regulated by the culture condition of the parental cells, starvation, and/or CDM with melatonin stimulations could subsequently impact the anti-inflammatory properties via multiple cell pathways.

### 2.5. Anti-Oxidative and Anti-Apoptosis Effects of EVs

**Figure 5:**
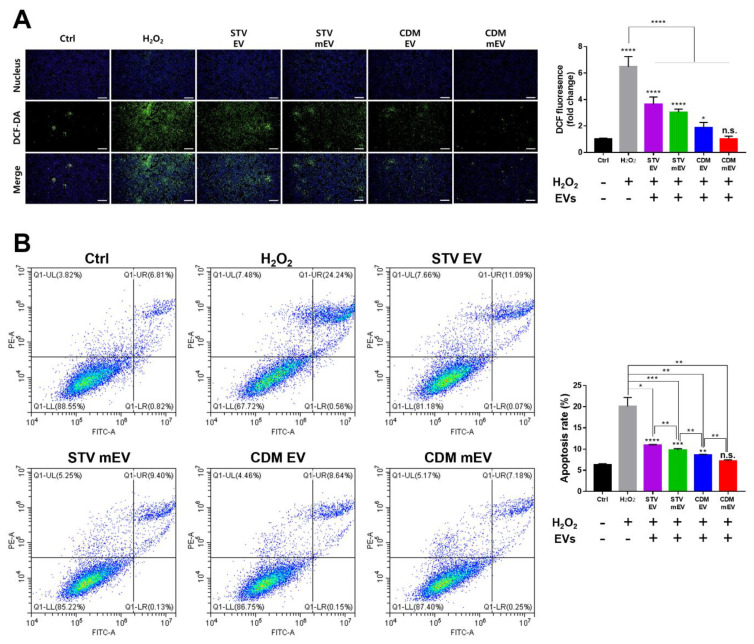
The bioactivities of EVs in ROS scavenging and anti-apoptosis in HK2 cells. (**A**) The ICC analysis for ROS scavenging activities of EVs and quantitative analysis with fluorescence intensities of DCF-DA using Image J. Scale bars equal to 500 μm. (**B**) The representative plots of flow cytometry analysis for PI/Annexin V of H_2_O_2_-pretreated HK2 cells with EVs, and the calculated apoptosis rate of EVs. Values are presented as mean ± SD (*n* = 3) and statistical significance was obtained with one-way analysis of ANOVA with Tukey’s multiple-comparison post-test (* *p* < 0.05; ** *p* < 0.01; *** *p* < 0.001; **** *p* < 0.0001).

The reactive oxygen species (ROS), one of critical pro-inflammatory factors, can induce macrophage activation, significantly elevating the inflammatory response in cells and tissues [49]. Additionally, the imbalance between ROS production and scavenging-indued cell dysfunction and tissue injury. With the anti-inflammatory and attenuating ROS properties of MSC-derived EVs, EVs have been treated to reduce oxidative stresses [50]. To assess the ROS scavenging capacity of EVs due to the affiliation of Apelin and AMPK signaling pathways to antioxidant effects in KEGG analysis, the cellular ROS assay was impelled with DCF-DA. The oxidative stress environment was induced by H_2_O_2_ treatment followed by the incubation of EVs. The results display that the damaged group had the highest DCF-DA intensity, as expected, whereas the CDM mEV group had a dramatically lower intensity that was not statistically significant in comparison to the control group (Figure 5A). These findings indicate that the ROS scavenging properties were maximized in CDM mEVs. In particular, apoptosis is implicated in a variety of diseases, including Alzheimer’s disease [51] and GVHD [52]. Moreover, many studies have identified the relations between ROS and apoptosis [53,54], which can be modulated by MSC-derived EVs [55]. As shown in the GO-BP term referring to the “Negative regulation of neuron apoptotic process” and the Apelin signaling pathway of the KEGG in bioinformatics analysis, hUCMSC-derived EVs were expected to be associated with anti-apoptosis. Following the consequence of the bioinformatics analysis, an apoptosis assay was carried out to clarify the anti-apoptosis properties of EVs (Figure 5B). The graph generated via quantified flow cytometry evaluation described the gradual decrease in the apoptosis rate with CDM culturing and melatonin modulation. Moreover, the CDM mEVs displayed similar trends in the results on ROS scavenging and anti-apoptosis. We speculated that these outcomes were caused by a harmonized interaction between CDM and melatonin, which is known to prevent cell apoptosis [56].

## 3. Materials and Methods

### 3.1. Cell Culture

Human umbilical cord mesenchymal stem cells (hUCMSCs; CHA Biotech Co., Ltd., Seongnam, Republic of Korea) were cultured using α-MEM (NM; HyClone Laboratories, Logan, UT, USA) with 10% fetal bovine serum (FBS, HyClone laboratories, Logan, UT, USA) and 1% antibiotic-antimycotic solution (GIBCO, Grand Island, NY, USA) or CellCor^TM^ CD MSC media (CDM; Xcell Therapeutics, Seoul, Republic of Korea) containing 1% antibiotic-antimycotic solution for serum-free conditions. Human proximal tubular epithelial cells (HK2; Korean Cell Line Bank, Seoul, Republic of Korea) were cultured using RPMI 1640 (HyClone laboratories, Logan, UT, USA), supplemented with 10% FBS and 1% antibiotic-antimycotic solution. Human coronary artery endothelial cells (HCAECs; Lonza, MD, USA) were cultured using the endothelial growth medium-2 bullet kit (EGM-2 MV bullet kit, CC-3202, Lonza, MD, USA). All cell types were incubated at 37 °C in a humidified environment with 5% CO_2_.

### 3.2. Isolation and Characterization of Extracellular Vesicles (EVs)

The hUCMSCs were cultured using two types of media, α-MEM (NM) and CellCor™ CD MSC media (CDM), for comparative analysis depending on the cell culture conditions. To make the conditions for starvation during EV isolations in NM (STV EVs), 10% FBS-supplemented α-MEM were replaced with phenol red-free DMEM (GIBCO, Grand Island, NY, USA) without FBS. The conditions for CDM were then maintained during isolations of EVs (CDM EVs). To isolate melatonin-stimulated EVs from phenol red-free DMEM (STV mEVs) and CDM culturing cells (CDM mEVs), the melatonin (1 μM, Sigma-Aldrich, St. Louis, MO, USA) was pretreated to cell culture media before collecting conditioned media. The conditioned media were collected four times to maximize the production yield of EVs. The collected cell culture media were centrifuged at 1300 rpm for 3 min and then filtered using a 0.22 µm vacuum filter/storage bottle system to eliminate large particles, such as cells, cell debris, and apoptotic bodies. The tangential flow filtration (TFF; KR2i, Repligen, Waltham, MA, USA) system with a 500 kDa molecular weight cut-off filter was used for the EV isolation. Using MONO ZetaView^®^ (PMX-120, Particle Metrix, Meerbusch, Germany) with the 488 nm scatter mode, the size and number of isolated EVs were measured. To achieve accurate analysis, the parameters of sensitivity, shutter, minimum trace length, and cell temperature were set at 75, 100, 15, and 25 °C, respectively, for all samples. Transmission electron microscopy (TEM; Hitachi, H-7600, 80 kV, Tokyo, Japan) was used to clarify the morphology of EVs. The EV solution was placed to dry on a copper grid supported by a 150-meshed formvar/carbon grid (FCF150-CU, Electron Microscopy Sciences, Hatfield, PA, USA) before being stained with an EM Stain 336 solution (R1260D; Agar Scientific, Stansted, UK) for negative staining.

### 3.3. Western Blot Analysis

For parallel comparisons, the same numbers of EVs were loaded onto nitrocellulose (NC) membranes after being loaded with 10% sodium dodecyl sulfate-poly acrylamide gel electrophoresis (SDS-PAGE). The NC membrane was blocked using a TBST solution diluted in 5% skim milk. To confirm EV surface markers, primary antibodies against CD63 (Abcam, Cambridge, MA, USA), CD81 (Santa Cruz Biotechnology, Santa Cruz, CA, USA), and CD9 (Abcam, MA, USA) were incubated with the protein-transferred NC membranes. The HRP-linked secondary antibodies (Cell Signaling Technology, Danvers, MA, USA) were used after incubating of the primary antibodies. The membrane was prepared with the enhanced chemiluminescence solution (GE Healthcare, Wauwatosa, WI, USA) and then visualized with ChemiDoc^TM^ XRS+ and ImageLab software (Bio-Rad, Hercules, CA, USA).

### 3.4. Quantification of the miRNA in EVs

The RNAs were isolated using the TRIzol^TM^ reagent (Ambion Life Technology, Carlsbad, CA, USA) from EVs in accordance with the manufacturer’s recommendations. The NanoDrop^TM^ One (Thermo Fisher Scientific, Marietta, OH, USA) was used to evaluate the quantity of the RNAs. Following the manufacturer’s instructions, the synthesis of cDNA and the RT-qPCR process were used to quantify the expression of miRNAs using the Mir-X miRNA RT-qPCR SYBR Kit (Takara, Otsu, Japan). The following primers were used to start reactions with the QuantStudio 3 (Applied Biosystems, Foster City, CA, USA) (hsa-let-7b-5p: 5′-AGAGGTAGTAGGTTGCATAGTT-3′, hsa-miR-23a-3p: 5′-ATCACATTGCCAGGGATTTCC-3′, and hsa-miR-100-5p: 5′-AACCCGTAGATCCGAACTTGTG-3′). The quantification data were obtained utilizing the 2^−ΔΔCt^ method, with U6 snRNA serving as a reference.

### 3.5. Bioinformatics Analysis of the Internal miRNA in EVs

The public algorithms for online prediction tools, TargetScan 8.0 (www.targetscan.org, accessed on 27 October 2022) and miRWalk (http://mirwalk.umm.uni-heidelberg.de/, accessed on 27 October 2022), were utilized to predict the target genes of the three types of representative miRNAs (hsa-let-7b-5p, hsa-miR-23a-3p, and hsa-miR-100-5p). The targeting genes of miRNAs were analyzed with the database for annotation, visualization, and integrated discovery (DAVID; https://david.ncifcrf.gov/, accessed on 27 October 2022) to assess the gene ontology (GO) and KEGG pathways, which predict the functionality of EVs. miRNet 2.0 (https://www.mirnet.ca/, accessed on 27 October 2022) was used to build the network of miRNAs and targeted genes.

### 3.6. Tube Formation Assay

The Matrigel matrix^TM^ (Corning, NY, USA) was coated to a pre-chilled 24-well plate and then incubated at 37 °C for 1 h before seeding cells. The HCAECs were seeded onto Matrigel-coated wells at a density of 1 × 10^5^ cells/well. After 16 h, calcein AM (C1430, Thermo Fisher Scientific, Cincinnati, OH, USA) was used to stain the cells. Fluorescence microscopy (CKX53, OLYMPUS, Tokyo, Japan) was employed to visualize the tube formation of cells. With the images from tube formation assays, angiogenesis-related parameters were assessed using the angiogenesis analyzer plugin for Image J (Wayne Rasband, NIH, Bethesda, MD, USA).

### 3.7. Cell Migration Assay

The HK2 cells were seeded in 6-well plates at a density of 2 × 10^5^ cells/well until they formed a confluent monolayer. The cells in the middle of the wells were scratched with a sterilized 1 mL pipette tip before being treated with the same concentration of EVs (1 × 10^8^ particles/mL) from four different conditioned media (STV EVs, STV mEVs, CDM EVs, and CDM mEVs), before then being rinsed with phosphate-buffered saline (PBS) solutions. After a 24 h incubation, cell migration was observed using a fluorescence microscopy (CKX53, OLYMPUS, Tokyo, Japan). The percentage of the open area was calculated using the ImageJ (Wayne Rasband, NIH, Bethesda, MD, USA) plugin for the wound-healing tool.

### 3.8. Immunocytochemistry (ICC)

The HK2 cells were fixed in 4% paraformaldehyde for 20 min at room temperature and cell permeability increased with 0.2% Triton-x and dissolved in PBS solution for 10 min at the same temperature. The cells were blocked with 1% BSA, and were dissolved in PBS solutions and incubated with the primary antibody at 4 °C overnight, followed by treatment with the secondary antibody for 1 h at room temperature. For immunofluorescent labeling, the NF-κB primary antibody (SC-8008, 2 µg/mL, Santa Cruz Biotechnology, Dallas, CA, USA) and the donkey anti-rabbit IgG (H+L) highly cross-adsorbed alongside the secondary antibody with Alexa Fluor^TM^ 488 (A-21206, 200:1, Invitrogen, Carlsbad, CA, USA) were used. Using fluorescence microscopy (CKX53, OLYMPUS, Tokyo, Japan), images of the immunofluorescence were obtained after the nucleus was stained with Hoechst (62,249, 1 µg/mL, Thermo Fisher Scientific, Marietta, OH, USA).

### 3.9. RT-qPCR for Pro-Inflammatory Factors

The HK2 cells were seeded on 6-well plates at a density of 2 × 10^5^ cells/well. The TNF-α (100 ng/mL) and EVs (1 × 10^8^ particles/mL) were simultaneously treated to the cells for 24 h. To assess the level of inflammation by real-time quantitative PCR (RT-qPCR) after 24 h, the RNA was extracted using the AccuPrep^®^ Universal RNA Extraction Kit (Bioneer, Daejeon, Korea). The reverse transcription of extracted RNA to cDNA was carried out using the PrimeScript^TM^ RT reagent kit (Takara, Otsu, Japan). RT-qPCR was performed using the SYBR green PCR reagent mix (Applied Biosystems, Foster City, CA, USA). The following primers were used to start reactions with the QuantStudio 3 (Applied Biosystems, CA, USA) (NF-κB: forward, 5′-CGGGATGGCTTCTATGAGG-3′ and reverse, 5′-CTCCAGGTCCCGCTTCTT-3′; IL-6: forward, 5′-GATGAGTACAAAAGTCCTGATCCA-3′ and reverse, 5′-CTGCAGCCACTGGTTCTGT-3′; IL-8: forward, 5′-AGACAGCAGAGCACACAAGC-3′ and reverse, 5′-ATGGTTCCTTCCGGTGGT-3′; 18 s rRNA: forward, 5′-GCAATTATTCCCCATGAACG-3′ and reverse, 5′-GGGACTTAATCAACGCAAGC-3′). The data were quantified utilizing the 2^−ΔΔCt^ method with 18 s rRNAs serving as a reference.

### 3.10. Cellular ROS Assay with 2′,7′-Dichlorofluorescin Diacetate (DCF-DA)

The HK2 cells were seeded at 5 × 10^5^ cells/well on 6-well plates. The cells were pretreated with EVs (1 × 10^8^ particles/mL) for 2 h, and damaged with hydrogen peroxide (H_2_O_2,_ 0.5 mM, Sigma-Aldrich, St. Louis, MO, USA) to upregulate ROS levels for 2 h. The DCF-DA solution (ab113851; Abcam, Cambridge, MA, USA) was treated with an optimum concentration (20 μM) to make media colorless for 30 min, as per the manufacturer’s instructions. Then, the Hoechst (62,249, 1 µg/mL, Thermo Fisher Scientific, Marietta, OH, USA) was treated for 10 min to stain the nuclei.

### 3.11. Apoptosis Assay

The HK2 cells were seeded on 6-well plates at 2 × 10^5^ cells/well. Two hours after EV (1 × 10^8^ particles/mL) treatments, 0.5 mM of H_2_O_2_ was treated to the cells for 2 h. The FITC Annexin V Apoptosis Detection Kit I (556,547, BD Biosciences, San Jose, CA, USA) was used in accordance with the manufacturer’s instructions to estimate the rates of apoptosis using flow cytometry (CytoFLEX; Beckman Coulter, Brea, CA, USA).

### 3.12. Statistical Analysis

GraphPad Prism 7 software was used to evaluate every statistical analysis. One-way analyses of variance (ANOVA) with Tukey’s multiple-comparison post-test were conducted to identify group differences. A *p* value of less than 0.05 was used to determine statistical significancy (* *p* < 0.05; ** *p* < 0.01; *** *p* < 0.001; **** *p* < 0.0001).

## 4. Conclusions

As an alternative to cell-based therapeutics, there have been numerous approaches to treat diseases, including COVID-19-induced sepsis, chronic kidney disease (CKD), and acute kidney injury (AKI), with controlling bioactivities of EVs. To regulate the functionalities of EVs, cell culture condition has a critical role because the characteristics of EVs represent the state of parental cells. In our previous study, we suggested the chemically defined media (CDM) for promoting the production yield and purity of EVs without serum components. In addition to overcoming a major hurdle for clinical uses of EVs with the utilization of CDM, the melatonin-pretreated method, one of the endogenous engineerings to produce functionally improved EVs, was further introduced. To demonstrate the improved bioactivities of EVs derived from melatonin-stimulated hUCMSCs in CDM, the expression levels of internal miRNAs were comparatively analyzed in four different types of EVs (STV EVs, STV mEVs, CDM EVs, and CDM mEVs), depending on the cell culture conditions. Moreover, among various types of EVs from hUCMSCs, the CDM mEVs showed a particularly superior effect on regenerative properties, such as wound healing, angiogenesis, anti-inflammation, ROS scavenging, and anti-apoptosis. Interestingly, the effects for the melatonin stimulations maximized in hUCMSCs were cultured with CDM as compared to a starvation condition. It is believed that our strategy to improve the production yield, purity, and functionalities of EVs could be actively utilized to treat various regenerative medicines.

## Data Availability

The datasets used and/or analyzed during the current study are available from the corresponding author on reasonable request.

## References

[1] Mazini L., Rochette L., Malka G. (2021). Exosomes Contribution in COVID-19 Patients’ Treatment. J. Transl. Med..

[2] Zhang B., Yeo R.W.Y., Lai R.C., Sim E.W.K., Chin K.C., Lim S.K. (2018). Mesenchymal Stromal Cell Exosome–Enhanced Regulatory T-Cell Production through an Antigen-Presenting Cell–Mediated Pathway. Cytotherapy.

[3] Munoz-Perez E., Gonzalez-Pujana A., Igartua M., Santos-Vizcaino E., Hernandez R.M. (2021). Mesenchymal Stromal Cell Secretome for the Treatment of Immune-Mediated Inflammatory Diseases: Latest Trends in Isolation, Content Optimization and Delivery Avenues. Pharmaceutics.

[4] Sengupta V., Sengupta S., Lazo A., Woods P., Nolan A., Bremer N. (2020). Exosomes Derived from Bone Marrow Mesenchymal Stem Cells as Treatment for Severe COVID-19. Stem Cells Dev..

[5] Fujii S., Miura Y., Fujishiro A., Shindo T., Shimazu Y., Hirai H., Tahara H., Takaori-Kondo A., Ichinohe T., Maekawa T. (2018). Graft-Versus-Host Disease Amelioration by Human Bone Marrow Mesenchymal Stromal/Stem Cell-Derived Extracellular Vesicles Is Associated with Peripheral Preservation of Naive T Cell Populations. Stem Cells.

[6] Wang Y., Yu D., Liu Z., Zhou F., Dai J., Wu B., Zhou J., Heng B.C., Zou X.H., Ouyang H. (2017). Exosomes from Embryonic Mesenchymal Stem Cells Alleviate Osteoarthritis through Balancing Synthesis and Degradation of Cartilage Extracellular Matrix. Stem Cell Res. Ther..

[7] Chen Y., Zhu Q., Cheng L., Wang Y., Li M., Yang Q., Hu L., Lou D., Li J., Dong X. (2021). Exosome Detection via the Ultrafast-Isolation System: EXODUS. Nat. Methods.

[8] Kim J.Y., Rhim W.K., Yoo Y.I., Kim D.S., Ko K.W., Heo Y., Park C.G., Han D.K. (2021). Defined MSC Exosome with High Yield and Purity to Improve Regenerative Activity. J. Tissue Eng..

[9] Kim J.Y., Rhim W.K., Seo H.J., Lee Y.J., Park C.G., Han D.K. (2021). Comparative Analysis of MSC-Derived Exosomes Depending on Cell Culture Media for Regenerative Bioactivity. Tissue Eng. Regen. Med..

[10] Haraszti R.A., Miller R., Stoppato M., Sere Y.Y., Coles A., Didiot M.C., Wollacott R., Sapp E., Dubuke M.L., Li X. (2018). Exosomes Produced from 3D Cultures of MSCs by Tangential Flow Filtration Show Higher Yield and Improved Activity. Mol. Ther..

[11] Gonzalez-King H., García N.A., Ontoria-Oviedo I., Ciria M., Montero J.A., Sepúlveda P. (2017). Hypoxia Inducible Factor-1α Potentiates Jagged 1-Mediated Angiogenesis by Mesenchymal Stem Cell-Derived Exosomes. Stem Cells.

[12] Sung D.K., Sung S.I., Ahn S.Y., Chang Y.S., Park W.S. (2019). Thrombin Preconditioning Boosts Biogenesis of Extracellular Vesicles from Mesenchymal Stem Cells and Enriches Their Cargo Contents via Protease-Activated Receptor-Mediated Signaling Pathways. Int. J. Mol. Sci..

[13] Noronha N.D.C., Mizukami A., Caliári-Oliveira C., Cominal J.G., Rocha J.L.M., Covas D.T., Swiech K., Malmegrim K.C.R. (2019). Priming Approaches to Improve the Efficacy of Mesenchymal Stromal Cell-Based Therapies. Stem Cell Res. Ther..

[14] Tocharus C., Puriboriboon Y., Junmanee T., Tocharus J., Ekthuwapranee K., Govitrapong P. (2014). Melatonin Enhances Adult Rat Hippocampal Progenitor Cell Proliferation via ERK Signaling Pathway through Melatonin Receptor. Neuroscience.

[15] Chen K., Tong C., Yang J., Cong P., Liu Y., Shi X., Liu X., Zhang J., Zou R., Xiao K. (2021). Injectable Melatonin-Loaded Carboxymethyl Chitosan (CMCS)-Based Hydrogel Accelerates Wound Healing by Reducing Inflammation and Promoting Angiogenesis and Collagen Deposition. J. Mater. Sci. Technol..

[16] Gómez-Florit M., Ramis J.M., Monjo M. (2013). Anti-Fibrotic and Anti-Inflammatory Properties of Melatonin on Human Gingival Fibroblasts in Vitro. Biochem. Pharmacol..

[17] Yea J.H., Yoon Y.M., Lee J.H., Yun C.W., Lee S.H. (2021). Exosomes Isolated from Melatonin-Stimulated Mesenchymal Stem Cells Improve Kidney Function by Regulating Inflammation and Fibrosis in a Chronic Kidney Disease Mouse Model. J. Tissue Eng..

[18] Han Y.S., Yoon Y.M., Go G., Lee J.H., Lee S.H. (2020). Melatonin Protects Human Renal Proximal Tubule Epithelial Cells against High Glucose-Mediated Fibrosis via the Cellular Prion Protein-Tgf-β-Smad Signaling Axis. Int. J. Med. Sci..

[19] Marino A., Battaglini M., Desii A., Lavarello C., Genchi G., Petretto A., Ciofani G. (2021). Liposomes Loaded with Polyphenol-Rich Grape Pomace Extracts Protect from Neurodegeneration in a Rotenone-Based in Vitro Model of Parkinson’s Disease. Biomater. Sci..

[20] Takhtfooladi H., Takhtfooladi M., Moayer F., Mobarakeh S. (2015). Melatonin Attenuates Lung Injury in a Hind Limb Ischemia-Reperfusion Rat Model. Rev. Port. Pneumol..

[21] Zhou L., Zhao D., An H., Zhang H., Jiang C., Yang B. (2015). Melatonin Prevents Lung Injury Induced by Hepatic Ischemia-Reperfusion through Anti-Inflammatory and Anti-Apoptosis Effects. Int. Immunopharmacol..

[22] Li Z., Nickkholgh A., Yi X., Bruns H., Gross M.L., Hoffmann K., Mohr E., Zorn M., Büchler M.W., Schemmer P. (2009). Melatonin Protects Kidney Grafts from Ischemia/Reperfusion Injury through Inhibition of NF-KB and Apoptosis after Experimental Kidney Transplantation. J. Pineal Res..

[23] Théry C., Witwer K.W., Aikawa E., Alcaraz M.J., Anderson J.D., Andriantsitohaina R., Antoniou A., Arab T., Archer F., Atkin-Smith G.K. (2018). Minimal Information for Studies of Extracellular Vesicles 2018 (MISEV2018): A Position Statement of the International Society for Extracellular Vesicles and Update of the MISEV2014 Guidelines. J. Extracell. Vesicles.

[24] Rikkert L.G., Nieuwland R., Terstappen L.W.M.M., Coumans F.A.W. (2019). Quality of Extracellular Vesicle Images by Transmission Electron Microscopy Is Operator and Protocol Dependent. J. Extracell. Vesicles.

[25] Park S.-Y., Kim D.-S., Kim H.-M., Lee J.-K., Hwang D.-Y., Kim T.-H., You S., Han D.K. (2022). Human Mesenchymal Stem Cell-Derived Extracellular Vesicles Promote Neural Differentiation of Neural Progenitor Cells. Int. J. Mol. Sci..

[26] Aday S., Hazan-Halevy I., Chamorro-Jorganes A., Anwar M., Goldsmith M., Beazley-Long N., Sahoo S., Dogra N., Sweaad W., Catapano F. (2021). Bioinspired Artificial Exosomes Based on Lipid Nanoparticles Carrying Let-7b-5p Promote Angiogenesis in Vitro and in Vivo. Mol. Ther..

[27] Hu H., Dong L., Bu Z., Shen Y., Luo J., Zhang H., Zhao S., Lv F., Liu Z. (2020). MiR-23a-3p-Abundant Small Extracellular Vesicles Released from Gelma/Nanoclay Hydrogel for Cartilage Regeneration. J. Extracell. Vesicles.

[28] Liu Z., Yang Y., Ju J., Zhang G., Zhang P., Ji P., Jin Q., Cao G., Zuo R., Wang H. (2022). MiR-100-5p Promotes Epidermal Stem Cell Proliferation through Targeting MTMR3 to Activate PIP3/AKT and ERK Signaling Pathways. Stem Cells Int..

[29] Li Z., Li Q., Tong K., Zhu J., Wang H., Chen B., Chen L. (2022). BMSC-Derived Exosomes Promote Tendon-Bone Healing after Anterior Cruciate Ligament Reconstruction by Regulating M1/M2 Macrophage Polarization in Rats. Stem Cell Res. Ther..

[30] Wu J., Kuang L., Chen C., Yang J., Zeng W.N., Li T., Chen H., Huang S., Fu Z., Li J. (2019). MiR-100-5p-Abundant Exosomes Derived from Infrapatellar Fat Pad MSCs Protect Articular Cartilage and Ameliorate Gait Abnormalities via Inhibition of MTOR in Osteoarthritis. Biomaterials.

[31] Moparthi L., Koch S. (2019). Wnt Signaling in Intestinal Inflammation. Differentiation.

[32] Reis M., Liebner S. (2013). Wnt Signaling in the Vasculature. Exp. Cell Res..

[33] Helker C.S.M., Eberlein J., Wilhelm K., Sugino T., Malchow J., Schuermann A., Baumeister S., Kwon H.B., Maischein H.M., Potente M. (2020). Apelin Signaling Drives Vascular Endothelial Cells towards a Pro-Angiogenic State. eLife.

[34] Schuman E.M., Madison D.V. (1994). Nitric Oxide and Angiogenesis. Am. J. Physiol..

[35] Nagata D., Mogi M., Walsh K. (2003). AMP-Activated Protein Kinase (AMPK) Signaling in Endothelial Cells Is Essential for Angiogenesis in Response to Hypoxic Stress. J. Biol. Chem..

[36] Meadows K.N., Bryant P., Pumiglia K. (2001). Vascular Endothelial Growth Factor Induction of the Angiogenic Phenotype Requires Ras Activation. J. Biol. Chem..

[37] Wu D., He L., Chen L. (2014). Apelin/APJ System: A Promising Therapy Target for Hypertension. Mol. Biol. Rep..

[38] Zeng X.J., Zhang L.K., Wang H.X., Lu L.Q., Ma L.Q., Tang C.S. (2009). Apelin Protects Heart against Ischemia/Reperfusion Injury in Rat. Peptides.

[39] Clevers H., Loh K.M., Nusse R. (2014). An Integral Program for Tissue Renewal and Regeneration: Wnt Signaling and Stem Cell Control. Science.

[40] Zhou F., Wang M., Ju J., Wang Y., Liu Z., Zhao X., Yan Y., Yan S., Luo X., Fang Y. (2019). Schizandrin a Protects against Cerebral Ischemia-Reperfusion Injury by Suppressing Inflammation and Oxidative Stress and Regulating the AMPK/Nrf2 Pathway Regulation. Am. J. Transl. Res..

[41] Xie L., Overbeek P.A., Reneker L.W. (2006). Ras Signaling Is Essential for Lens Cell Proliferation and Lens Growth during Development. Dev. Biol..

[42] Molina J.R., Adjei A.A. (2006). The Ras/Raf/MAPK Pathway. J. Thorac. Oncol..

[43] Nour S., Imani R., Chaudhry G.R., Sharifi A.M. (2021). Skin Wound Healing Assisted by Angiogenic Targeted Tissue Engineering: A Comprehensive Review of Bioengineered Approaches. J. Biomed. Mater. Res.—Part A.

[44] DiPietro L.A. (2016). Angiogenesis and Wound Repair: When Enough Is Enough. J. Leukoc. Biol..

[45] Lee Y.Y., Park H.H., Park W., Kim H., Jang J.G., Hong K.S., Lee J.Y., Seo H.S., Na D.H., Kim T.H. (2021). Long-Acting Nanoparticulate DNase-1 for Effective Suppression of SARS-CoV-2-Mediated Neutrophil Activities and Cytokine Storm. Biomaterials.

[46] Ko K.-W., Park S.-Y., Lee E.H., Yoo Y.-I., Kim D.-S., Kim J.Y., Kwon T.G., Han D.K. (2021). Integrated Bioactive Scaffold with Polydeoxyribonucleotide and Stem-Cell-Derived Extracellular Vesicles for Kidney Regeneration. ACS Nano.

[47] Yang G.H., Lee Y.B., Kang D., Choi E., Nam Y., Lee K.H., You H.J., Kang H.J., An S.H., Jeon H. (2021). Overcome the Barriers of the Skin: Exosome Therapy. Biomater. Res..

[48] Kim S., Kim Y., Hyun Y.S., Choi H., Kim S.Y., Kim T.G. (2021). Exosomes from Human Cord Blood Plasma Accelerate Cutaneous Wound Healing by Promoting Fibroblast Function, Angiogenesis, and M2 Macrophage Differentiation. Biomater. Sci..

[49] Xiong H., Wang S., Sun Z., Li J., Zhang H., Liu W., Ruan J., Chen S., Gao C., Fan C. (2022). The ROS-responsive Scavenger with Intrinsic Antioxidant Capability and Enhanced Immunomodulatory Effects for Cartilage Protection and Osteoarthritis Remission. Appl. Mater. Today.

[50] Hong Y., Sun Y., Rong X., Li D., Lu Y., Ji Y. (2020). Exosomes from Adipose-Derived Stem Cells Attenuate UVB-Induced Apoptosis, ROS, and the Ca2+ Level in HLEC Cells. Exp. Cell Res..

[51] Xu X., Lai Y., Hua Z.C. (2019). Apoptosis and Apoptotic Body: Disease Message and Therapeutic Target Potentials. Biosci. Rep..

[52] Wang L., Romero M., Ratajczak P., Lebœuf C., Belhadj S., Peffault De Latour R., Zhao W.L., Socié G., Janin A. (2013). Increased Apoptosis Is Linked to Severe Acute GVHD in Patients with Fanconi Anemia. Bone Marrow Transplant..

[53] Simon H.U., Haj-Yehia A., Levi-Schaffer F. (2000). Role of Reactive Oxygen Species (ROS) in Apoptosis Induction. Apoptosis.

[54] Hwang Y.H., Kim Y.J., Lee D.Y. (2021). Hepatic and Renal Cellular Cytotoxic Effects of Heparin-Coated Superparamagnetic Iron Oxide Nanoparticles. Biomater. Res..

[55] Yan Y., Jiang W., Tan Y., Zou S., Zhang H., Mao F., Gong A., Qian H., Xu W. (2017). HucMSC Exosome-Derived GPX1 Is Required for the Recovery of Hepatic Oxidant Injury. Mol. Ther..

[56] Zhao X.M., Hao H.S., Du W.H., Zhao S.J., Wang H.Y., Wang N., Wang D., Liu Y., Qin T., Zhu H. (2016). Bin Melatonin Inhibits Apoptosis and Improves the Developmental Potential of Vitrified Bovine Oocytes. J. Pineal Res..

